# Association between low-frequency ultrasound and hip fractures - comparison with DXA-based BMD

**DOI:** 10.1186/1471-2474-15-208

**Published:** 2014-06-16

**Authors:** Mikko Määttä, Petro Moilanen, Jussi Timonen, Pasi Pulkkinen, Raija Korpelainen, Timo Jämsä

**Affiliations:** 1 Department of Medical Technology, University of Oulu, Institute of Biomedicine, PO Box 5000, FI-90014 Oulu, Finland; 2 Infotech Oulu, University of Oulu, Oulu, Finland; 3 Department of Physics, University of Jyväskylä, Jyväskylä, Finland; 4 Department of Sports and Exercise Medicine, Oulu Deaconess Institute, Oulu, Finland; 5 Institute of Health Sciences, University of Oulu, Oulu, Finland; 6 Medical Research Center Oulu, Oulu University Hospital and University of Oulu, Oulu, Finland; 7 Department of Diagnostic Radiology, Oulu University Hospital, Oulu, Finland

**Keywords:** Quantitative ultrasound, Speed of sound, Osteoporosis, Hip fracture, Cortical bone

## Abstract

**Background:**

New methods for diagnosing osteoporosis and evaluating fracture risk are being developed. We aim to study the association between low-frequency (LF) axial transmission ultrasound and hip fracture risk in a population-based sample of older women.

**Methods:**

The study population consisted of 490 community-dwelling women (78–82 years). Ultrasound velocity (V_LF_) at mid-tibia was measured in 2006 using a low-frequency scanning axial transmission device. Bone mineral density (BMD) at proximal femur measured using dual-energy x-ray absorptiometry (DXA) was used as the reference method. The fracture history of the participants was collected from December 1997 until the end of 2010. Lifestyle-related risk factors and mobility were assessed at 1997.

**Results:**

During the total follow-up period (1997–2010), 130 women had one or more fractures, and 20 of them had a hip fracture. Low V_LF_ (the lowest quartile) was associated with increased hip fracture risk when compared with V_LF_ in the normal range (Odds ratio, OR = 3.3, 95% confidence interval (CI) 1.3-8.4). However, V_LF_ was not related to fracture risk when all bone sites were considered. Osteoporotic femoral neck BMD was associated with higher risk of a hip fracture (OR = 4.1, 95% CI 1.6-10.5) and higher risk of any fracture (OR = 2.4, 95% CI 1.6-3.8) compared to the non-osteoporotic femoral neck BMD. Decreased V_LF_ remained a significant risk factor for hip fracture when combined with lifestyle-related risk factors (OR = 3.3, 95% CI 1.2-9.0).

**Conclusion:**

Low V_LF_ was associated with hip fracture risk in older women even when combined with lifestyle-related risk factors. Further development of the method is needed to improve the measurement precision and to confirm the results.

## Background

Osteoporotic fractures possess a significant public health problem that is increasing due to aging population. At the moment, the golden standard used in fracture risk assessment is bone mineral density (BMD) measurements using dual energy x-ray absorptiometry (DXA). Recently, World Health Organization (WHO) introduced FRAX®, a fracture risk calculator that combines easily obtained clinical information and DXA-based femoral neck BMD, if available, to estimate the 10-year osteoporotic fracture probability [1].

Quantitative ultrasound (QUS) has raised interest as an alternative method to x-ray-based imaging for measuring bone status. There is a number of different QUS methods that have been used to assess bone status and to evaluate fracture risk [2]. The advantages of ultrasound include a relatively low cost and a portable technique with no ionizing radiation. The results of QUS measurements have been shown to be associated with fracture risk [3]. A question has arisen whether QUS parameters can serve as a surrogate for DXA-based BMD to improve FRAX® estimate [4].

A novel low-frequency (LF) axial transmission QUS method for assessing the properties of cortical bone has been shown to reflect bone density and cortical thickness [5-7]. A relationship between the LF tibial velocity (V_LF_) and geometry of proximal femur in older women has also been suggested [8]. However, the suitability of the method for individual fracture risk assessment has not yet been established.

In this study we evaluated the association between the LF axial transmission ultrasound method and fractures in a population-based sample of older women, using the standard DXA as the reference method. We hypothesized that decreased ultrasound velocity measured on tibia is associated with increased hip fracture risk in elderly females. In our previous population based prospective study we assessed life-style related determinants of hip fracture in elderly females [9]. Here we analyzed how the combination of low-frequency ultrasound and previously determined lifestyle-related factors are related to the risk of hip fracture.

## Methods

### Subjects and clinical assessment

The study population consisted of 490 women born between 1924 and 1927, originally recruited in 1997 as a population-based cohort to study the risk factors for osteoporosis and fractures [10]. All those still alive belonging to the original cohort were invited to clinical measurements in 2006. A total of 618 women attended these measurements, and 490 were measured using quantitative ultrasound. The fracture history between December 1, 1997 and December 31, 2010 was collected from hospital discharge registers. The fractures were confirmed manually from medical records to avoid the bias of recording multiple hospitalizations due to a single fracture. Health and lifestyle information, including medical history, age at menopause, smoking habits, alcohol and coffee consumption, physical activity and mobility, calcium and vitamin D intakes, and fracture history was collected at study baseline in 1997 using self-administrative questionnaires and interviews. The assessment of functional mobility at baseline was done using the “Timed Up & Go” (TUG) test [11]. All subjects gave a written informed consent and the study protocol was approved by the Ethics Committee of the Northern Ostrobothnia Hospital District. The study was done in accordance with the Declaration of Helsinki.

### Quantitative ultrasound measurements

The speed of sound was recorded in the medial mid-shaft of the left tibia using a scanning low-frequency (LF) axial transmission device. The principle of the device and the measurement setup has been published previously [7,8]. In brief, two separate transducers (*f*_*c*_ = 200 kHz) mounted on a rail scanned a 30 mm distance. The time of flight of the first arriving signal (FAS) was determined using the first maximum. The apparent velocity of the FAS (V_LF_) was determined by measuring the time of flight for a number of source-receiver distances. The precision error was characterized by CVrms which was 3.2%. The standardized coefficient of variation (SCV) [12] of the method was 6.6%. Due to the lack of established reference population data, we used the present study population to define the range of SCV.

### DXA measurements

A Hologic DXA device (Delphi QDR series, Hologic, Bedford, MA, USA) was used to get the reference data. Standard anteroposterior positioning was used to measure the femoral neck bone mineral density (BMD) of the left proximal femur.

### Statistical analysis

**Figure 1:**
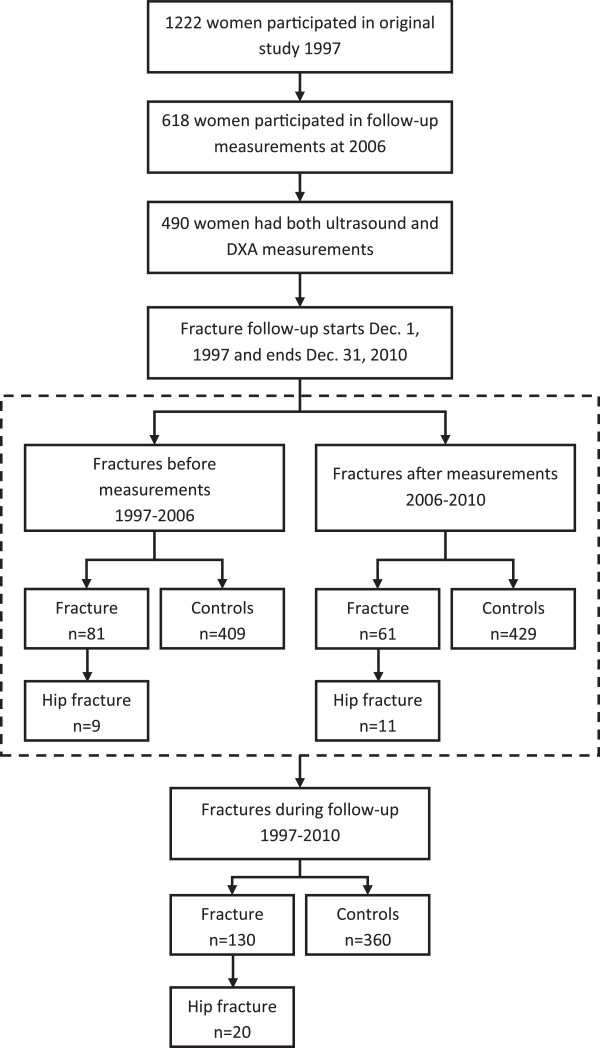
**Consort chart.** Number of women with and without fractures at different timepoints.

The subjects of the study were divided into three groups based on their fracture history (Figure 1): a) women without fractures (NF, controls), b) women with any fracture (Fx), and c) women with a hip fracture (Hip Fx). All the hip fracture patients were also included in group (b). The fracture data of three different time periods were analyzed: a) from the beginning of the follow-up period until the time of bone measurements (1997–2006), b) from the time of the bone measurements until the end of the follow-up period (2006–2010), and c) the whole follow-up period (1997–2010). The women with fractures were compared to those without fractures. Since the data were normally distributed the independent samples *t*-test was used to analyze the statistical significance of the differences between the NF, Fx, and Hip Fx groups. The study subjects were also classified in to normal, osteopenic, and osteoporotic according to their femoral neck BMD T-score in accordance with the WHO definition [13]. Due to the lack of an established reference population, T-scores could not be calculated for low-frequency ultrasound velocity (V_LF_). Thus, V_LF_ results were divided into three groups based on quartiles within the study population: a) low V_LF_ (0-25%), b) moderate V_LF_ (25-50% and 50-75% combined), and c) high V_LF_ (75-100%). Crosstabulation and the χ^2^ test were used to compare the distribution of the subjects with fractures and those without fractures within these groups. To further analyze the statistical significance of the association of bone measurement results (QUS/DXA) with the fracture risk, a multivariate logistic regression analysis was used. The results of those analyses are reported as the odds ratios (OR) and 95% confidence intervals (CI). The imaging modality (QUS/DXA) used was included in the model and the forward stepwise (likelihood ratio) method was used to form the final models. All models were adjusted with age and BMI. For the fractures that occurred after the measurements in 2006, the predictive ability of each bone measurement value was assessed using the Cox proportional hazards model and the corresponding hazard ratios (HR), and the 95% CIs were calculated. A similar protocol to logistic regression was used. In addition, previous fractures (1997–2006) (yes, no) was added to models as covariates. The follow-up time between the measurement time and the time of the first fracture, death, or end of the follow-up period was recorded. Logistic regression models were also calculated to analyze the effect of combining V_LF_ and previously determined [9] lifestyle-related risk factors for hip fracture. In our previous population based cohort study BMI, functional mobility, physical activity, hypertension, coffee consumption, and daily smoking predicted hip fractures in 1222 old women. These factors were used as a covariates to determine the best fit model for the current subpopulation with ultrasound and DXA measurements.

Statistical analyses were done using SPSS for Windows (Release 18.0, SPSS Inc., Chicago, IL, USA). In all tests, p-values less than 0.05 were considered statistically significant.

## Results

During the first follow-up period (1997–2006), 98 fractures occurred in 81 women (Figure 1, Table 1). Between 2006 and 2010, 61 subjects sustained fractures; in total 76 different fractures occurred. Altogether, between the years 1997 and 2010, 174 different fractures occurred in 130 subjects. Wrist fracture was the most common fracture (n = 51). There were 9 hip fractures between 1997 and 2006, and 11 hip fractures after the 2006 measurements, totaling in 20 hip fractures during the whole follow-up period. Thirty-five subjects died during the follow-up period.

**Table 1:** Distribution of fractures (n (%)) according to the ICD-10 code with selected subdivisions in a population-based sample of older women (n = 490)

		**1997–2006**	**2006–2010**	**1997–2010**
M80	Osteoporosis with pathological fracture		2 (2.6)	2 (1.1)
S12	Fracture of neck		1 (1.3)	1 (0.6)
S22	Fracture of rib(s), sternum and thoracic spine	3 (3.1)	3 (3.9)	6 (3.4)
S32	Fracture of lumbar spine and pelvis			
S32.0	Fracture of lumbar vertebra		9 (11.8)	9 (5.2)
	Other	3 (3.1)	4 (5.3)	7 (4.0)
S42	Fracture of shoulder and upper arm	11 (11.2)	6 (7.9)	17 (9.8)
S52	Fracture of forearm			
S52.5	Fracture of lower end of radius	29 (29.6)	22 (28.9)	51 (29.3)
	Other	5 (5.1)	1 (1.3)	6 (3.4)
S62	Fracture at wrist and hand level	7 (7.1)	5 (6.6)	12 (6.9)
S72	Fracture of femur			
S72.0	Fracture of neck of femur	4 (4.1)	7 (9.2)	11 (6.3)
S72.1	Pertrochanteric fracture	1 (1.0)	4 (5.3)	5 (2.9)
S72.2	Subtrochanteric fracture	4 (4.1)		4 (2.3)
	Other	3 (3.1)	6 (7.9)	9 (5.2)
S82	Fracture of lower leg, including ankle	26 (26.5)	5 (6.6)	31 (17.8)
S92	Fracture of foot, except ankle	2 (2.0)	1 (1.3)	3 (1.7)
Total number of fractures		98 (100)	76 (100)	174 (100)

ICD-10 = International Classification of Diseases, 10^th^ revision. Time periods represent previous fractures (1997–2006) and future fractures (2006–2010) with respect to the bone measurements, and all fractures during the follow-up period (1997–2010).

The characteristics of different fracture groups are shown in Table 2. The women with a hip fracture were taller than the women without fractures (p < 0.05). Those with a previous fracture (1997–2006) had a lower V_LF_ than the ones without fractures (p < 0.05). Subjects who sustained a fracture during the follow-up period had a lower femoral neck BMD than those in the NF group (p < 0.05), except when comparing the Hip Fx 1997–2006 and NF groups.

**Table 2:** Characteristics of the women with different fracture status (NF, Fx, and Hip Fx) in different time periods (1997–2006, 2006–2010, and 1997–2010) at the time of the measurements (2006) (n = 490)

	**NF**		**Fx 1997-2010**		**Hip Fx 1997-2010**	
	**n =360**		**n = 130**		**n = 20**	
			**Fx 1997-2006**	**Fx 2006-2010**	**Hip Fx 1997-2006**	**Hip Fx 2006-2010**
			**n = 81**	**n = 61**	**n = 9**	**n = 11**
Age [years]	79.9 (1.2)		80.0 (1.2)		80.0 (1.3)	
			79.7 (1.2)	80.3 (1.1)^*^	79.3 (1.4)	80.5 (0.9)
Weight [kg]	67.7 (11.3)		67.8 (12.2)		69.8 (15.7)	
			69.5 (12.3)	65.8 (12.2)	75.7 (10.2)^*^	65.0 (18.2)
Height [cm]	155.2 (5.4)		155.6 (5.8)		158.9 (7.2)^*^	
			156.6 (5.4)^*^	154.5 (6.4)	160.7 (4.4)^*^	157.4 (8.8)
BMI [kg/m^2^]	28.1 (4.5)		28.0 (4.7)		27.4 (4.6)	
			28.4 (4.8)	27.5 (4.6)	29.3 (3.6)	25.9 (4.9)
V_LF_ [m/s]	3583 (193)		3547 (200)		3515 (245)	
			3534 (204)^*^	3564 (203)	3500 (291)	3527 (215)
Femoral neck BMD [g/cm^2^]	0.654 (0.102)		0.606 (0.083)^*^		0.589 (0.102)^*^	
			0.609 (0.078)^*^	0.605 (0.096)^*^	0.611 (0.089)	0.570 (0.112)^*^

Values are Mean (SD).

^*^Independent samples *t*-test p-value < 0.05 when compared to NF group.

Differences between distributions in the NF and Hip Fx groups were observed in both V_LF_ and femoral neck BMD (p < 0.05) (Table 3). Also, there was a difference in the distribution of subjects based on femoral neck BMD between the Fx and NF groups.

**Table 3:** **The distribution of women with and without a fracture and hip fracture according to the V**_**LF **_**and BMD values**

		**NF**	**Fx 1997–2010**	**Hip Fx 1997–2010**
		**(n = 360)**	**(n = 130)**	**(n = 20)**
V_LF_	Highest 25%	101 (28)	24 (18)	5 (25)^*^
	25% to 75%	170 (47)	69 (53)	5 (25)
	Lowest 25%	89 (25)	37 (28)	10 (50)
Femoral neck BMD	Normal	73 (20)	11 (8)^*^	2 (10)^*^
	Osteopenic	206 (57)	65 (50)	8 (40)
	Osteoporotic	81 (23)	54 (42)	10 (50)

Values are n (%). ^*^Distribution of the group is different than that of the NF group (p-value of χ^2^ test < 0.05).

Based on the regression analysis, low V_LF_ was not associated with previous fractures (1997–2006) or with the fractures that occurred after the measurements (2006–2010) (Table 4). Decreased femoral neck BMD, however, was associated with increased fracture risk in all follow-up periods; estimated risks (odds and hazard ratios) varied between 1.8 and 2.5 compared to the women with femoral neck T-score > -2.5.

**Table 4:** **Association of low V**_**LF **_**and osteoporotic femoral neck BMD with fractures that occurred during different follow-up periods**

			**Fx 1997-2006**		**Fx 2006-2010**		**Fx 1997-2010**	
			**NF n = 409, Fx n = 81**		**NF n = 429, Fx n = 61**		NF n = 334, Fx n = 121*	
		**n**	**OR (95% CI)**	**p**	**HR (95% CI)**	**p**	**OR (95% CI)**	**p**
V_LF_	Moderate or high (25-100%)	364						
	Low (0-25%)	126	1.6 (0.9 - 2.6)	0.088	0.8 (0.4 - 1.4)	0.359	1.3 (0.8 - 2.0)	0.326
Femoral neck BMD	T-score > -2.5	355						
	T-score ≤ -2.5	135	1.8 (1.1 - 3.0)	0.019	2.4 (1.4 - 3.9)	0.001	2.5 (1.6 - 3.9)	<0.001

All regression models were adjusted by age and BMI. In Cox regression previous fracture (during 1997–2006) was also used as a covariate. NF = non-fractured group, Fx = fractured group, n = number of subjects, OR = odds ratio of logistic regression, HR = hazard ratio of Cox regression, CI = confidence interval, p = p-value of the covariate in the model. *Subjects who died during the follow-up were excluded (NF n = 26, Fx n = 9).

Decreased V_LF_ was associated with an increased risk of hip fracture occurred before the measurements (1997–2006) (OR = 6.3; Table 5). Additionally, across the whole follow-up period (1997–2010), low V_LF_ was associated with higher risk of hip fracture compared to moderate or high V_LF_ (OR = 3.3). An osteoporotic femoral neck BMD predicted hip fractures after the measurements (2006–2010) with a hazard ratio (HR) of 4.8 (95% CI 1.4-16.6) for subjects with a T-score ≤ -2.5 compared to the women with normal or osteopenic T-score. Low femoral neck BMD was also associated with an increased risk of hip fracture (OR = 4.1, 95% CI 1.6-10.5) during the whole follow-up period (1997–2010) compared to women with normal or osteopenic femoral neck BMD. When V_LF_ was combined with previously assessed [9] hip fracture risk factors regression model for the same population (Table 6), the final model included V_LF_, low physical activity, and impaired functional mobility (p = 0.001, Table 6), V_LF_ being the strongest independent factor for hip fracture (OR = 3.3, 95% CI 1.2–9.0). Femoral neck BMD did not reach statistical significance when included in the regression analyses along with lifestyle-related risk factors.

**Table 5:** **Association of low V**_**LF **_**and osteoporotic femoral neck BMD with hip fractures that occurred during different follow-up periods**

		**Hip Fx 1997-2006**			**Hip Fx 2006-2010**			**Hip Fx 1997-2010**		
		**NF n = 409, Hip Fx n = 9**			**NF n = 429, Hip Fx n = 11**			NF n = 334, Hip Fx n = 19*		
		**n**	**OR (95% CI)**	**p**	**n**	**HR (95% CI)**	**p**	**n**	**OR (95% CI)**	**p**
V_LF_	Moderate or high (25-100%)	313			324			260		
	Low (0-25%)	105	6.3 (1.5 - 25.5)	0.010	116	1.7 (0.5 - 5.7)	0.415	93	3.3 (1.3 - 8.4)	0.012
Femoral neck BMD	T-score > -2.5	311			325			267		
	T-score ≤ -2.5	107	1.5 (0.4 - 6.0)	0.593	115	4.8 (1.4 - 16.6)	0.013	86	4.1 (1.6 – 10.5)	0.003

All regression models were adjusted by age and BMI. In Cox regression previous fracture (during 1997–2006) was also used as a covariate. NF = non-fractured group, Hip Fx = hip fracture group, n = number of subjects, OR = odds ratio of logistic regression, HR = hazard ratio of Cox regression, CI = confidence interval, p = p-value of the covariate in the model. *Subjects who died during the follow-up were excluded (NF n = 26, Hip Fx n = 1).

**Table 6:** **Logistic regression models without and with V**_**LF **_**for having a hip fracture in a population-based sample of older women**

	**OR**	**(95% CI)**	**p-value**
Lifestyle-related risk factors^1^			0.002^*^
TUG ≥ 11 s vs. less (referent)	3.4	(1.2 - 9.9)	0.026
Low PA vs. moderate to high (referent)	2.8	(1.0 - 7.5)	0.046
Coffee consumption > 3 cups/day vs. less (referent)	0.3	(0.1 - 1.0)	0.051
Lifestyle-related risk factors and V_LF_^2^			0.001^*^
Low V_LF_ (0-25%) vs. Moderate or high (25-100%) (referent)	3.3	(1.2 - 9.0)	0.018
Low PA vs. moderate to high (referent)	3.1	(1.1 - 8.5)	0.028
TUG ≥ 11 s vs. less (referent)	3.1	(1.0 - 8.9)	0.042

Odds ratios (OR) are calculated compared to the NF group. TUG “Timed Up & Go” test, PA physical activity, CI confidence interval. The number of subjects in the analyses was Hip Fx n = 18, NF n = 296. ^1^Age, BMI, TUG, PA, hypertension, coffee consumption, and smoking were included in the analysis to form the best model, ^2^The best model after including V_LF_ in the analysis, ^*^p-value for the full model.

## Discussion

In this population-based study, we used a low-frequency ultrasound scanner to assess the association between decreased ultrasound velocity and fracture risk. To our knowledge, this is the first time that low-frequency axial transmission ultrasound is being used in a population-based cohort of older women. The reported results show that decreased ultrasound velocity (V_LF_) was associated with higher risk of hip fracture compared to moderate or high V_LF_. DXA and LF ultrasound yielded similar results when comparing the hip fracture and non-fracture groups.

Our findings are in line with earlier studies on the fracture-discrimination ability of similar axial transmission ultrasound devices. Using the Omnisense (Sunlight, BeamMed Ltd, Petah Tikva, Israel) device, operating at a center frequency of 1.25 MHz, Nguyen et al. [14] reported that decreased tibial SOS is associated with increased fracture risk independently of BMD and age with an odds ratio (OR) of 1.75. The corresponding OR as determined for the femoral neck BMD here was 2.11. Damilakis et al. [15] found no difference using the Omnisense device in the tibial SOS of healthy subjects and subjects with osteoporotic fracture. Nevertheless, they reported increased osteoporotic fracture ORs for ultrasound measurements on the radius and phalanx (ORs between 1.7 and 2.7). In other studies with an Omnisense device, SOS measured on the radius has been shown to discriminate subjects with hip fracture from controls with no fractures (ORs varying between 1.9 and 2.7) [16-18]. Another axial transmission QUS device, Myriad Soundscan (Myriad Ultrasound systems, Israel) uses a center frequency of 250 kHz which is closer to that used in this study. Using this device, Stegman et al. [19] reported a low-energy appendicular fracture OR of 1.4 for the tibial SOS. This observation is in good agreement with the present study, where we found a similar trend in the retrospective part of the study. Augat et al. [20] found an increased OR of 1.7 for the tibial SOS of subjects who sustained hip fracture compared to non-fracture controls. The corresponding OR for the femoral neck BMD was 3.5 in their study. This SOS OR is somewhat lower than those observed in the present study for hip fractures. Also, Talmant et al. [21] studied the fracture discrimination ability of a 1 MHz bidirectional ultrasonometer. They reported a fracture OR of 1.8 for the radius SOS and of 2.1 for the femoral neck BMD. Direct comparison between the ultrasound methods used is difficult due to differences in the ultrasound methodology and measurement setup. Also, the bone site and type of fracture, as well as the study populations, affect the results. However, the majority of the studies imply that axial transmission ultrasound is capable of assessing fracture risk.

Some studies have assessed the combining clinical risk factors and QUS parameters [22-24]. In the current study low V_LF_ remained a significant risk factor for hip fracture when included in the lifestyle-related risk factor model observed in our previous study with the same population [9]. However, the lifestyle-related risk factors were collected at baseline whereas QUS measurements were made at eight years from baseline. Thus, the results need to be confirmed in future prospective studies.

In the present study ultrasound velocity was measured at mid-tibia. Even though osteoporosis is a systemic disease, the degree of changes in bone structure varies between bone sites. In weight-bearing sites (e.g. the tibia) bone loss is not as big as in non-weight-bearing sites (e.g. the radius and phalanx) [25]. Thus, even if the tibia is an easily accessible site for axial ultrasound measurements, it may not be the most responsive site to osteoporotic changes. Recently, we have reported that LF axial-transmission ultrasound in the radius is able to retrospectively discriminate postmenopausal women with fractures from age-matched controls with no fractures using an improved version of the LF ultrasound device used here [26].

The strength of the present study was the use of a population-based cohort of older women. The other strength was the relatively long follow-up time before and after the bone measurements. However, this study also had some limitations. One major limitation was the limited measurement precision of the current prototype device. The bulky mechanical scanning setup limits the positioning accuracy and does not enable proper correction for the impacts of overlying soft tissue [8]. As a result, the *in vivo* precision (CV_rms_) was limited. During the follow-up period of the study, significant mechanical improvements were implemented in the device, and a CV of 0.5% could be achieved with the latest device version [5,26]. Obviously, this valuable information available after the device improvement cannot retrospectively improve the quality of the results presented here. The other limitation is the retrospective nature of the study. It is possible that the fracture events before the measurements caused the changes in bone properties (e.g. via altered loading conditions) and affected on outcome of the measurement. Also, the number of hip fractures was relatively low, which significantly limited the statistical reliability of the results. Additionally, a minor limitation was the lack of reference data for young and healthy population, which disabled the determination of the T-score. Instead, we used quartiles to define the subjects with low V_LF_ values.

## Conclusions

In conclusion, decreased low-frequency ultrasound velocity was associated to increased hip fracture risk despite the limited measurement precision. The results reported here can be used to further improve the measurement precision of the method so as to reliably predict future fractures. To this end carefully planned follow-up studies are needed.

## Competing interests

The authors declare that they have no competing interests.

## Authors’ contributions

MM participated in the fracture data collection, carried out the main analyses, and drafted the manuscript. PM participated in the analyses and interpretation of ultrasound data and manuscript writing. JT and PP made a contribution to the design of the study and revising the manuscript. RK and TJ participated in study conception and design, data interpretation and made substantial contributions to the manuscript. RK also participated significantly to collection of lifestyle-related risk factors data. All authors have read, revised, and given their final approval of the version to be published.

## Pre-publication history

The pre-publication history for this paper can be accessed here:

http://www.biomedcentral.com/1471-2474/15/208/prepub
